# COVID-19, the Future Vaccine and What It Means for Cancer Patients on Immunotherapy

**DOI:** 10.3389/fonc.2020.631611

**Published:** 2021-02-01

**Authors:** Karim Hussien El-Shakankery, Joanna Kefas, Rowan Miller

**Affiliations:** ^1^ Department of Medicine, St Bartholomew’s Hospital, London, United Kingdom; ^2^ Department of Medical Oncology, St Bartholomew’s Hospital, London, United Kingdom

**Keywords:** vaccination, COVID-19, coronavirus, cancer, immunotherapy, immune checkpoint inhibitors, immunosuppression

## Introduction

As of 22^nd^ December 2020, the United Kingdom (UK) reported 2,110,314 cases of coronavirus disease (COVID-19) with 79,349 deaths (1). There have been subsequent critical changes in cancer services, under the presumption that these patients are at higher risk. Data supporting this is predominantly from the initial peak of the outbreak and limited to small retrospective studies from China, the United States and Italy. The UK coronavirus cancer monitoring project (CCMP), launched in March, is the largest prospective database of COVID-19 infections in oncology patients. Despite reporting a 28% mortality rate, the CCMP largely attributes this to advancing age, male gender, and non-cancer comorbidities (2). A recent meta-analysis of 15 studies by Zhang et al. echoed these findings and prognostic indicators, noting an overall cancer fatality rate of 22.4% and fatality rate of 24.3% in patients specifically undergoing immunotherapy (3). As COVID-19 cases rise exponentially once more, we brace another wave of a disease that is both highly virulent and highly unpredictable. Even with lessons learned from recent months and novel effective treatments, we still face unmeasurable challenges associated with protecting vulnerable groups. During this first wave, ‘shielding’ in the UK enabled us to minimize exposure to cancer patients undergoing systemic anti-cancer treatments (SACTs). Focusing resources on the current pandemic comes at the cost of ethical rationing, diagnostic delays, treatment delays and suspension of most clinical trials. The full fallout of which will show itself with time.

## The Role of T-Cells in Vaccination, Immunotherapy, and COVID-19

The global research shift toward COVID-19 means that at present there are over 100 COVID-19 vaccines in development, with over 50 of these undergoing clinical trials (4). Over eight different vaccination approaches are under investigation including weakened or inactivated forms, viral vector, nucleic acid and protein-based types. Certainly, large multi-center trials have already reported encouraging results, as discussed below. T-cells play a vital role in the recognition of foreign antigens and development of such immunity. Importantly, T-cells also play a key role in immunosurveillance of cancer cells, and their manipulation forms the fundamental bases of anti-cancer-targeted immunotherapy.

In recent years, immune checkpoint inhibitors (ICIs) have revolutionized the management of solid tumors. ICIs augment T-cell mediated anti-tumor responses, most commonly *via* programmed cell death 1 (PD-1) and cytotoxic T-lymphocyte-associated antigen-4 (CTLA-4) pathways (5). By facilitating host immune clearance of malignant cells, ICIs provide effective treatment options in the absence of harmful myelosuppression. However, with their increasing use comes growing evidence of immune related adverse events (irAEs). Gambichler et al. (6) comprehensively summarized available information relating to the controversial use of ICIs in cancer patients with viral infections, including COVID-19. Evidence shows ICIs should not be considered highly immunosuppressive and their use is not an independent risk factor for COVID-19 susceptibility. In fact, just as there are known similarities between immune responses to cancer and other viral infections, similarities have also been drawn between immune checkpoint pathways and COVID-19 immunogenicity. COVID-19 cases show lymphopenia is a predictor of mortality and T lymphocyte exhaustion is a distinctive feature, likely influenced by the high levels of PD-1 expression observed. Gambichler et al. (6) go on to hypothesize that ICIs may even benefit cancer patients who acquire COVID-19 during treatment, though this has not been observed or shown in case reports. The potential benefits or detriments of ICI use in this context are further complicated by the clinical and pathological similarities between irAEs and COVID-19 autoreactivity. COVID-induced cytokine storms, myocarditis and pneumonitis are all reported, adding further diagnostic uncertainties.

## Immunotherapy Use During the COVID-19 Pandemic

With much still to learn about the pathogenicity of the novel COVID-19 infection and its complex relationship with ICIs, we must continue to cautiously balance the risk-benefit of ICI use in vulnerable cancer patients. Currently, the European Society for Medical Oncology (ESMO) consensus is to continue ICI use, even in COVID-19 positive patients, if there is survival benefit (7). However, an international survey of oncologists identified a lack of consensus, and evidence of caution and reserve when dealing with initiating and maintaining such treatment in these current circumstances (8).

## The COVID-19 Vaccination

In late 2020, the UK government secured a supply of vaccines from Pfizer–BioNTech (nucleoside-modified RNA vaccine), Oxford/AstraZeneca (inactivated virus vaccine) and 6 other manufacturers. To date, only the Pfizer–BioNTech immunization has been approved for use in the UK, and the vaccination program has already begun through hospital hubs in specific geographical areas which are not yet nationwide (9–11). Phase 3 trials of the Pfizer–BioNTech and Oxford/AstraZeneca vaccines have reported both these immunizations to be well tolerated and effective in healthy individuals (12, 13). However, exclusion criteria resulted in patients with a diagnosis of malignancy and patients undergoing cytotoxic/immunosuppressive treatments for cancer, being excluded from both trials. As an exception, the Oxford/AstraZeneca vaccine trial accepted patients with skin basal cell carcinoma and cervical carcinoma *in situ* only (12, 13). Trials for other vaccines in development, such as the Moderna vaccine, also do not include cancer patients undergoing treatment (14). To our knowledge, there are currently no trials investigating coronavirus vaccine use specifically in immunosuppressed patients, though there are ongoing studies investigating the efficacy of long-acting monoclonal antibody infusions as a vaccination alternative (NCT04625972). Due to worldwide demand and expedited vaccine production processes, the longevity of immunity and long-term safety profile of all vaccines under development remains to be established.

Although vaccination programs are underway, significant demand worldwide for such a vaccine has resulted in the need for prioritization criteria by the UK Joint Committee on Vaccination and Immunization (JCVI) (9). This criteria is primarily based on age being the single most important risk factor for COVID-19 mortality, and healthcare workers being at the highest risk of acquiring the infection and transmitting it to vulnerable others. Resultantly, the presence of cancer or other vulnerable co-morbidities does not carry a high priority (**Table 1**) (9). Though countries such as France have adopted similar prioritization criteria to the UK, various other European countries and Australia have considered immunosuppressed and cancer patients as equal priority to those of older age (15–17).

**Table 1:** UK Joint Committee on Vaccination and Immunization COVID-19 Vaccination Priority List (9).

Priority	Prioritization Criteria for the COVID-19 Vaccine
1	Care Home Residents and Carers
2	Those aged >80 years Health and social care workers
3	Those aged >75 years
4	Those aged >70 years Extremely vulnerable adults of any age
5	Those aged >65 years
6	Those aged 16-65 years with comorbidities that label them vulnerable
7	Those aged >60 years
8	Those aged >55 years
9	Those aged >50 years

Though these trial results are encouraging, they are representative of a healthy and ideal cohort. Questions remain to be answered regarding vaccine safety and efficacy for cancer patients and those on immunotherapies; this is especially relevant in patients undergoing T-cell mediated SACTs. Furthermore, the recent discovery of a new coronavirus variant, identified by the UK COVID-19 Genomics Consortium, brings further uncertainty. Of the 17 changes and mutations the new variant harbors, one particular mutation in the virus’ spike protein conveys augmented virulence and increased risk of transmission. Though it is suspected that this mutated spike protein will not alter vaccine efficacy, work is still underway to definitively conclude this (18).

## The Relationship Between ICI Use and Other Viral Infections

Although there are uncertainties regarding COVID vaccination in cancer patients, it is also vital to highlight that little is known about the efficacy of other existing and commonly used vaccinations in those with malignancy. Current UK guidance strongly advises against live vaccinations for all patients undergoing SACTs (19). Such strong recommendations on vaccination (live or inactivated) in patients with cancer are supported by low-quality evidence, and data is certainly lacking regarding their efficacy and interaction with ICIs. Administration of the inactivated influenza vaccine in patients treated with ICIs is the most researched example. Studies show sustained protective titres in these patients, compared to the diminished responses observed in those treated with cytotoxic chemotherapy (20). Reassuringly, this response was paired with no significant increase in irAEs, therefore routine influenza vaccination is encouraged.

As widespread ICI use occurred after the 2012 Middle East respiratory syndrome coronavirus (MERS-CoV) and 2003 severe acute respiratory syndrome (SARS-CoV) outbreaks, studies investigating the interaction between immunotherapy and these coronaviruses are not available. In addition, as neither viruses have effective vaccinations against them, it is not known what effects immunization may have had on cancer patients and service delivery. Although we have high hopes for the COVID-19 vaccine, we must consider the possibility that it may be poorly tolerated and/or accepted by the general public once widely available, and may display altered or inadequate responses specifically in cancer patients on ICIs.

## Looking Ahead

Regardless of if vaccination is effective or not, cancer services must continue. Even prior to the worst winter the NHS will face in all of its 72 years, there was already concerning evidence of the consequences of diagnostic and treatment delays, cancer screening suspensions, and a decline in ‘two-week wait’ referrals (21). While we wait for the vaccine program to fully roll out and prove effective, cancer services must adapt in the best way they can, balancing exposure risk with continued service delivery. They must continue to develop effective targeted strategies including changes seen so far with home treatment options, virtual/remote assessments and COVID-19-secure hospital zones.

COVID-19 has already caused extensive disruption to cancer patients throughout 2020. Despite the lack of objective evidence regarding the vaccine’s efficacy in ICI patients, oncologists must encourage this cohort to take part in the vaccination program, trusting the positive data reported in COVID-19 vaccine trials, drawing on their previous experiences, and extrapolating previous data published concerning existing vaccination programs. Mindful of the high risk of severe COVID-19 infection and death in cancer patients, a consensus statement by ESMO also actively encourages vaccination. Having considered the risks and benefits, they recommend that those with cancer should be prioritized for vaccination regardless of comorbidity and age, stressing that those currently in clinical trials should not be exempt (17).

A forward plan of action, at both a national policy and local service level, is imperative. Throughout, centers must prepare to analyze prospective data, share knowledge and co-operate to ensure timely responses to any beneficial or detrimental observations. We must collaborate to obtain key data on COVID infections in cancer patients, interactions between COVID and treatments (including ICIs), vaccine efficacy in the context of cancer- or treatment-mediated immunosuppression, and vaccine safety in this specific cohort. As the UK vaccination program uses non-live immunizations and only excludes those who are pregnant and those less than 16 years of age, cancer patients are not, and should not be exempt (9). If the COVID-19 vaccine proves to be everything we hope it to be, there is light at the end of the tunnel.

## Data Availability Statement

Data sharing is not applicable to this article as no new data were created or analyzed in this study.

## Author Contributions

All contributors meet authorship criteria and have contributed to the manuscript equally. All authors contributed to the article and approved the submitted version.

## Conflict of Interest

The authors declare that the research was conducted in the absence of any commercial or financial relationships that could be construed as a potential conflict of interest.

## Abbreviations

ICIs, Immune checkpoint inhibitors; UK, United Kingdom; COVID-19, Coronavirus disease; CCMP, UK coronavirus cancer monitoring project; SACTs, Systemic anti-cancer treatments; PD-1, Programmed cell death 1; CTLA-4, Cytotoxic T-lymphocyte-associated antigen-4 pathways; irAEs, Immune related adverse events; ESMO, European Society for Medical Oncology; JCVI, UK Joint Committee on Vaccination and Immunization; MERS-CoV, 2012 Middle East respiratory syndrome coronavirus; SARS-CoV, 2003 severe acute respiratory syndrome.
